# Mitochondrial Transplantation Promotes Remyelination and Long-Term Locomotion Recovery following Cerebral Ischemia

**DOI:** 10.1155/2022/1346343

**Published:** 2022-09-15

**Authors:** Tao Chen, Yuanyuan Zhu, Jia Jia, Han Meng, Chao Xu, Panpan Xian, Zijie Li, Zhengang Tang, Yin Wu, Yan Liu

**Affiliations:** ^1^Branch of Cerebral Vascular Diseases, Department of Neurosurgery, General Hospital of Southern Theater Command, The First School of Clinical Medicine, Southern Medical University, PLA, No. 111, Liuhua Road, Guangzhou, 510515 Guangdong, China; ^2^Institute of Neurology, Renmin Hospital, Hubei University of Medicine, Shiyan, Hubei, China; ^3^Department of Neurobiology and Institute of Neurosciences, School of Basic Medicine, Fourth Military Medical University, 169 Chang Le Xi Road, Xi'an, Shaanxi, China; ^4^Department of Gastroenterology, Renmin Hospital, Hubei University of Medicine, Shiyan, Hubei, China; ^5^Department of Pharmacy, Xi'an Gaoxin Hospital, No. 16, Tuanjie Road, Hi-Tech Zone, Xi'an, Shaanxi 710075, China; ^6^Department of Neurology, Foresea Life Insurance Guangzhou General Hospital, Guangzhou, Guangdong, China

## Abstract

Cerebral ischemia usually leads to axonal degeneration and demyelination in the adjacent white matter. Promoting remyelination still remains a challenging issue in the field. Considering that ischemia deprives energy supply to neural cells and high metabolic activities are required by oligodendrocyte progenitor cells (OPCs) for myelin formation, we assessed the effects of transplanting exogenous healthy mitochondria on the degenerating process of oligodendrocytes following focal cerebral ischemia in the present study. Our results showed that exogenous mitochondria could efficiently restore the overall mitochondrial function and be effectively internalized by OPCs in the ischemic cortex. In comparison with control cortex, there were significantly less apoptotic and more proliferative OPCs in mitochondria-treated cortex. More importantly, higher levels of myelin basic protein (MBP) and more morphologically normal myelin-wrapped axons were observed in mitochondria-treated cortex at 21 days postinjury, as revealed by light and electron microscope. Behavior assay showed better locomotion recovery in mitochondria-treated mice. Further analysis showed that olig2 and lipid synthesis signaling were significantly increased in mitochondria-treated cortex. In together, our data illustrated an antidegenerating and myelination-promoting effect of exogenous mitochondria, indicating mitochondria transplantation as a potentially valuable treatment for ischemic stroke.

## 1. Introduction

Stroke is one of the leading causes of disability and dementia around the world [1]. Approximately 80% of stroke is cerebral ischemia, which usually leads to permanent loss of neurons and oligodendrocytes in the injury area. In the past decades, numerous researches focused on and developed various treatments for neuroprotection [2, 3]. Relatively, there still lacks effective strategy for preventing the degeneration of oligodendrocytes and demyelination following ischemia.

One pathological feature of cerebral ischemia is the disruption of energy supply, which usually leads to mitochondria dysfunction [4]. Mitochondria damage is thought as the major cause of cell death and oxidative stress [5]. Restoring or promoting mitochondria function has been regarded as promising for reducing the secondary injury following ischemia [6]. Recently, *in vivo* transplantation of healthy mitochondria has been proposed as an effective way to boost mitochondria function and exert neuroprotection [7, 8]. For example, mitochondrial transplantation exerted remarkable beneficial effects in preventing secondary injury in traumatic brain injury and cognitive decline in aging [9, 10]. High metabolic requirement was observed during the myelination process of oligodendrocyte precursor cells (OPCs) [11]. Whether mitochondria transplantation could exert protective effects on oligodendrocyte lineage cells remains interesting to be explored.

In this study, we investigated this issue by evaluating the survival and proliferation of oligodendrocyte progenitor cells (OPCs) and myelination in ischemic cortex following mitochondria transplantation.

## 2. Methods

### 2.1. Photochemical Ischemia Model

Adult C57BL/6J male mice (body weight: 20-23 g) with 8-10 weeks of age were adopted. All mice were specific pathogen-free and housed under 12 light/12 dark cycle, controlled temperature (22-24°C) with free access to water and standard rodent chaw. All animal experiments were carried out according to the protocols approved by the Animal Care and Use Committees of Fourth Military Medical University (license number: 20211024). Focal cortical ischemia was induced as described with minor modification [12]. Rose bengal (Sigma) was injected i.v. at 20-25 mg/kg. A skull window was made ranging from 0.4 to 2.4 mm anterior to the Bregma and 0.7 to3.2 mm right to the midline. The brain was illuminated for 15-18 minutes using a cold light source (Zeiss FL2000 LCD).

### 2.2. Mitochondria Preparation and ATP Measurement

Mitochondria were purified from allogeneic mouse liver as described [10]. Liver tissue was homogenated on ice by mitochondria isolation buffer, and the tissue lysate was centrifuged at 1000 g for 5-8 min. The supernatant was centrifuged at 3500 g for 12 min. The resulting precipitate was resuspended with 15% Percoll and centrifuged at 21000 g to precipitate mitochondria. For mitochondria quantification, mtDNA was isolated and quantified as described [13]. Briefly, plasmids containing ND1 (a mitochondria gene) were diluted into graded concentrations (1, 10, 10^2^, 10^3^, 10^4^, 10^5^, 10^6^, 10^7^, and 10^8^ copies per ml) to make standard curve. qPCR was performed using the following ND1 primers. ND1-F: CCGAGCATCTTATCCACGCT; ND1-R: ATGGTGGTACTCCCGCTGTA. mtDNA copies were calculated according to the standard curve. Before transplantation, freshly isolated mitochondria were labeled with Mito-Tracker (Mito-Tracker Red CMXRos, M9940, Solarbio) and transplanted immediately after isolation. For mitochondrial transplantation, two bilateral injections with 1 mm distance to lesion center were made. Approximately 2 × 10^7^ mitochondria were injected for each site with 50 nl/min at the depth of 0.75 mm and 1.5 mm from dura mater, respectively.

For ATP measurement, brain tissues (including lesion center and brain regions 3 mm within the lesion boarder) were dissected and homogenized. ATP levels were measured by using an ATP assay kit (BC0300, Solarbio Life Sciences). The activity of mitochondrial complex was measured by using assay kits (BC1445, BC3240, BC0945, BC3235, BC0515, and BC0305, Solabio Life Sciences).

### 2.3. Immunohistochemistry

Animals were sacrificed, and serial sections (18-24 *μ*m in thickness) were prepared. Sections were blocked by PBS containing 0.3% Triton X-100 and 5% bovine serum albumin (BSA) for 1.5 h. Primary antibodies were incubated overnight as the followings: rabbit anti-NG2 antibody (1 : 200, Millipore), rabbit anti-CNPase (1 : 200, Millipore), rabbit anti-MBP antibody (1 : 200, GeneTex), rabbit anti-Ki67 antibody (1 : 200, GeneTex), rat anti-PDGFR*α*(1 : 500, Abcam), rabbit anticleaved caspase-3 (1 : 200, GeneTex), and goat anti-Sox10 antibody (1 : 100, Santa Cruz Biotech). After washing, sections were incubated with secondary antibodies conjugated with Alexa Fluor 488 (donkey anti-rabbit, 1 : 400, Molecular probes) or Alexa Fluor 594 (donkey anti-goat or anti-rat IgG, 1 : 800, Molecular probes) for 2-4 h at room temperature. The nuclei were stained by DAPI (1 : 1500, Sigma). Images were taken by using confocal microscope (FV3000, Olympus) with same setting to reduce variation.

### 2.4. Electron Microscopic Study

At 21 d after mitochondria transplantation, the mice were perfusion fixed with 2% glutaraldehyde. Sections (50 *μ*m in thickness) were prepared and fixed again with 1% osmium tetroxide. Then, sections were subsequently dehydrated with graded ethanol and embedded in Epon 812. Ultrathin sections were cut by using an LKB Nova Ultratome (Bromma). Final counterstaining was performed with uranyl acetate and lead citrate. After that, sections were observed, and images were taken by using a JEM-1230 electron microscope (JEM, Tokyo). The *g*-ratio was measured as the ratio of the inner to the outer radius of the myelin sheath of the cross section of axons as described [14].

### 2.5. Western Blotting

Ischemic cortex was dissected and homogenized. SDS-PAGE was conducted and protein transferred to PVDF membrane. Rabbit anti-MBP antibody (1 : 1000, GeneTex) and mouse anti-*β*-actin antibody (1 : 6000, Proteintech) were incubated with protein-loaded membrane at 4°C overnight. HRP-conjugated anti-rabbit or anti-mouse secondary antibodies (1 : 10000; Pierce) were incubated for 1 h at room temperature. Bands were visualized with an ECL kit (Pierce) and images analyzed by Image J.

### 2.6. Locomotion Assay

Glass sliding test was carried out at 1 d, 3 d, 7 d, and 21 d posttransplantation as described [15]. Mice were videotaped in glass cylinder for 15 min. The numbers of contacts and the numbers of sliding movements of each forelimb at the wall of the cylinder for every spontaneous stand-up were scored. The sliding index was calculated as: the number of sliding/(number of contact + number of sliding) × 100.

Rotarod test was conducted at 1 d, 3 d, 7 d, and 21 d posttransplantation. Mice were lowered onto the rotating roller, and the timer was started immediately upon the release of the tail. The rod was accelerated 5-50 rpm during the course of 5 min. The latency to fall in each trial was recorded. Three trials were performed daily for 3 days.

### 2.7. RNA Sequencing and Quantitative PCR

At 7 days after transplantation, injured tissue in the control mice and mitochondria-treated mice were dissected, and total RNA was isolated. After quality check, RNA sequencing was carried out by Gene Denovo Biotechnology Co., Ltd. (Guangzhou, China). For quantitative PCR, the following primers were used:

FABP-5-F: TGAAAGAGCTAGGAGTAGGACTG

FABP-5-R: CTCTCGGTTTTGACCGTGATG

FABP-7-F: GGACACAATGCACATTCAAGAAC

FABP-7-R: CCGAACCACAGACTTACAGTTT

Olig2-F: TCCCCAGAACCCGATGATCTT

Olig2-R: CGTGGACGAGGACACAGTC

FASN-F: GGAGGTGGTGATAGCCGGTAT

FASN-R: TGGGTAATCCATAGAGCCCAG

### 2.8. Statistical Analysis

At least 3 biological repeats were carried out for each immunohistochemistry and Western blotting experiment. For morphological quantification, all the double-stained cells in lesion area and in the adjacent region 300 *μ*m around the lesion boarder were counted from at least 6 sections of each mouse. The percentages were calculated by dividing the number of total OPCs with double-positive OPCs. For behavior assays, there were at least 10 mice in each group. The data were presented as means ± S.E. and analyzed by Student's *t* test or one-way ANOVA, followed by Dunnett post hoc [16]. *P* values less than 0.05 were considered as statistically significant. SPSS16.0 was adopted to perform statistical analysis.

## 3. Results

### 3.1. Internalization of Exogenous Mitochondria by Oligodendrocyte Progenitor Cells (OPCs) in Ischemic Cortex

**Figure 1:**
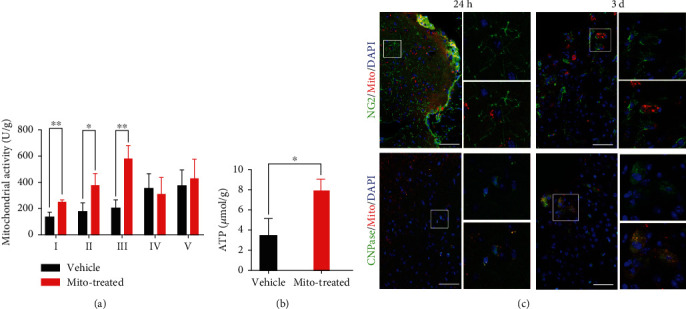
Restoration of mitochondrial function in ischemic cortex by exogenous mitochondria. (a) Mitochondrial complex activity in control and mitochondria-treated cortex. (b) ATP levels in control and mitochondria-treated cortex. (c) Combination of NG2 and CNPase staining with Mito-Tracker (Mito) labeling in ischemic cortex at 24 h and 3 d posttransplantation. Images in the frames were magnified. Notice the internalized mitochondria by NG2- and CNPase-positive cells. Notice the increase of mitochondrial complex activity and ATP level in mitochondria-treated mice. One way ANOVA for (a). Student's t test for (b). *n* = 5 mice per group in (a, b). *N* = 3 mice per group in (c). ^∗^*P* < 0.05. ^∗∗^*P* < 0.01. Bars = 50 *μ*m.

Previous studies have demonstrated dysfunction of mitochondria following ischemia [17]. We first explored if transplanting healthy mitochondria could enhance the oval mitochondria function in the ischemic cortex. As the liver is the organ contains the largest number of mitochondria, mitochondria were isolated from allogeneic liver as reported [10]. 2 × 10^7^ mitochondria were injected for each ischemic site. Three days later, the activity of complexes І, II, and III and the levels of ATP were increased significantly in mitochondria-treated cortex, as compared with vehicle-treated cortex (Figures 1(a) and 1(b)). Then, we explored whether OPCs could uptake exogenous mitochondria. Internalization of Mito-Tracker labeled mitochondria was evaluated at 24 h and 3 d following transplantation. At 24 h after grafting, approximately 58% NG2-positive cells (OPCs) and 40% CNPase-positive cells (immature oligodendrocytes) in a zone within 300 *μ*m from the margin of lesion boarder were Mito-Tracker positive. At 3 d postgrafting, approximately 52% NG2-positive cells and 43% CNPase-positive cells in this region were Mito-Tracker labeled (Figure 1(c)). No Mito-Tracker-labeled MBP-positive cells (mature oligodendrocytes) were detected (data not shown). These data indicated grafted mitochondria could be internalized efficiently by OPCs in ischemic cortex.

### 3.2. Promotion of OPC Survival and Proliferation by Exogenous Mitochondria

**Figure 2:**
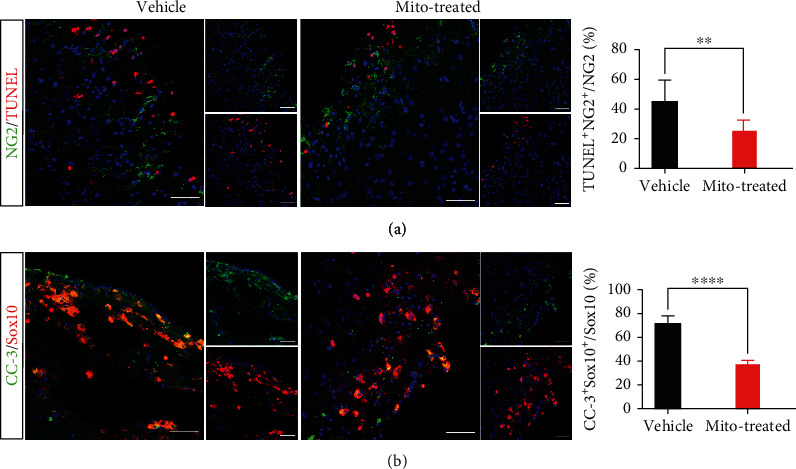
Effects of grafted mitochondria on the survival of OPCs. (a) Combination of TUNEL and NG2 staining at 3 d posttransplantation. (b) Double immunostaining of cleaved caspase-3 (CC-3) and Sox10 at 3 d posttransplantation. TUNEL/NG2- and CC-3/Sox10-positive cells were significantly decreased in mitochondria-treated mice, as compared to control. Representative images were taken from lesion boarder. Student's *t* test. *N* = 5 mice per group. ^∗∗^*P* < 0.01. ^∗∗∗^*P* < 0.001. ^∗∗∗∗^*P* < 0.0001. Bars = 50 *μ*m.

We next examined if exogenous mitochondria could prevent the death of OPCs. Combination of TUNEL and NG2-staining showed that the TUNEUL/NG2-positive cells in the ischemic region decreased by approximately 45% in mitochondria-grafted cortex at 3 d posttransplantation (Figure 2(a)). In addition, double immunostaining of cleaved caspase-3 (CC-3) with Sox10 (a marker of OPC) showed that CC-3/Sox10-positive cells reduced by approximately 49% at 3 d posttransplantation, as compared with that in vehicle control (Figure 2(b)). These data demonstrated that grafted mitochondria promoted the survival of OPCs in ischemic cortex.

**Figure 3:**
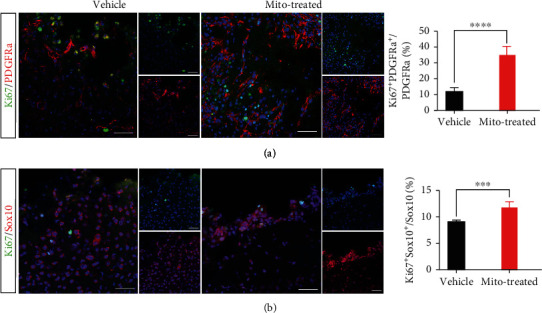
Effects of exogenous mitochondria on the proliferation of OPCs. (a) Double immunostaining of Ki67/PDGFR*α* at 5 d posttransplantation. (b) Double immunostaining of Ki67/Sox10 at 5 d posttransplantation. Ki67/PDGFR*α*- and Ki67/Sox10-positive cells significantly increased in mitochondria-treated mice, as compared to control. Representative images were taken from lesion boarder. Student's *t* test. *N* = 5 mice per group. ^∗∗^*P* < 0.01. ^∗∗∗^*P* < 0.001. ^∗∗∗∗^*P* < 0.0001. Bars = 50 *μ*m.

As the development of OPCs is closely associated with their internal metabolic condition, we assessed if mitochondria transplantation could affect the proliferation of OPCs. Double immunostaining of Ki67 (a marker of cell-cycle entry) with two OPC markers PDGFR*α* and Sox10 showed that there were significantly more Ki67/PDGFR*α*-positive and Ki67/Sox10-positive cells in mitochondria-treated cortex as compared with that of control group (Figures 3(a) and 3(b)).

### 3.3. Improvement of Myelination and Locomotion by Exogenous Mitochondria

**Figure 4:**
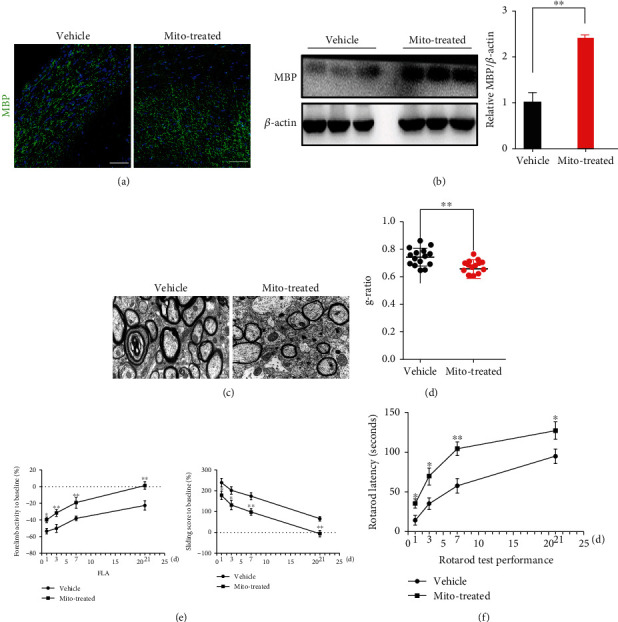
Effects of grafted mitochondria on myelination and locomotion recovery. (a, b) Immunostaining and Western blotting of MBP in control and mitochondria-treated mice at 3 w posttransplantation. Notice the higher levels of MBP expression in mitochondria-treated mice. (c, d) Representative EM images of myelin in control and mitochondria-treated mice at 3 w posttransplantation and *g*-ratio of myelin. Notice the loose and degenerating myelin in control group and the compact and morphologically normal myelin in mitochondria-treated group. (e) Forelimb activity (glass sliding) assay. (f) Rotarod assay. Notice the better locomotion recovery in mitochondria-treated mice. Student's *t* test for (b, d). One-way ANOVA for (e, f). *N* = 3 mice per group in (a–d). *N* = 10 mice per group in (e, f). ^∗^*P* < 0.05. ^∗∗^*P* < 0.01. Bars = 50 *μ*m in (a, b) and 500 nm in (c).

We then evaluated if mitochondria transplantation could attenuate the demyelination in ischemic cortex and the subcortical corpus callosum at 21 d posttransplantation. Both immunostaining and Western blotting demonstrated significantly higher levels of myelin basic protein (MBP) in mitochondria-treated cortex, as compared with vehicle control (Figures 4(a) and 4(b)). In addition, under electron microscope, demyelinated axons with thin and loose myelin could be frequently detected in vehicle-treated cortex, while more axons with thick and intact myelin were observed in mitochondria-treated cortex (Figure 4(c)). The *g*-ratio of myelin was significantly lower in the mitochondria-treated than that in vehicle-treated cortex (Figure 4(d)). These data indicated that mitochondrial transplantation was beneficial for myelin preservation in ischemic cortex.

In the end, we examined if mitochondria transplantation could affect the locomotion recovery after ischemia. Glass sliding assay which evaluates forelimb activity was conducted. From 3 d posttransplantation, the total activity of affected forelimbs significantly increased in mitochondria-treated group, as compared with that in vehicle-treated group (Figure 4(e)). Accordingly, the sliding activity of affected forelimb reduced significantly in mitochondria-treated group (Figure 4(e)). In addition, Rotarod assay which accesses motor coordination and balance demonstrated that mitochondria-treated mice exhibited significantly longer running time on the rod (Figure 4(f)). These data indicated that exogenous mitochondria was beneficial for promoting functional recovery of ischemic stroke.

### 3.4. Upregulation of Lipid Synthesis Signaling and Olig2 by Exogenous Mitochondria

**Figure 5:**
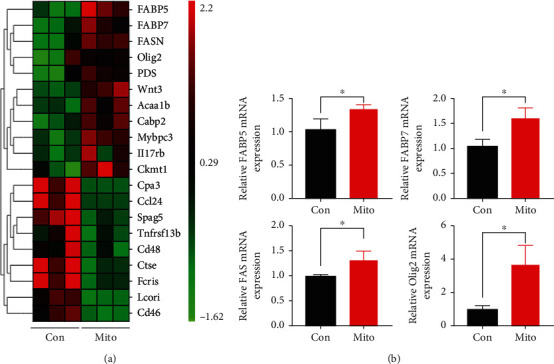
Transcriptome analysis of mitochondria-treated cortex and validation of top increased genes. (a) Heatmap of top changed genes. (b) qPCR validation of top increased genes. Notice that the mRNA levels of FABP5, FABP7, FASN, and Olig2 were significantly increased in mitochondria-treated cortex. Student's *t* test for (b). *N* = 3 mice per group. ^∗^*P* < 0.05.

To explore how exogenous mitochondria stimulated OPC proliferation and myelination, we performed transcriptome analysis of ischemic cortex treated with mitochondria or vehicle control at 7 days after transplantation. The results showed that 179 genes were upregulated and 196 genes downregulated in mitochondria-treated cortex, as compared to that of control. Among the top 10 increased genes, we noticed the presence of fatty acid binding protein 5 (FABP5), fatty acid binding protein 7 (FABP7), fatty acid synthase (FASN), Olig2, Wnt3, and acetyl-Coenzyme A acyltransferase 1B (Acaa1b) (Figure 5(a)). Reportedly, these genes are involved in multiple stages of OPC development [18–21]. We validated the expression of these genes by real-time RT-PCR. The results showed that the mRNA levels of FABP5, FABP7, FASN, and Olig2 were significantly increased in mitochondria-treated cortex (Figure 5(b)). As FABP5, FABP7, and FASN play important roles in lipid metabolism, these data indicated that exogenous mitochondria might stimulate remyelination via activating lipid synthesis signaling and Olig2-mediated gene transcription.

## 4. Discussion

In this study, we explored the effects of transplantation of exogenous mitochondria on the survival, proliferation, and myelin formation of OPCs. First, we demonstrated the efficient endocytosis of exogenous mitochondria by OPCs. By apoptosis and cell proliferation analysis, we revealed that mitochondria grafting promoted the survival and proliferation of OPCs. More importantly, our data showed that, mitochondria transplantation promoted remyelination in the ischemic cerebral cortex and facilitated long-term functional recovery.

As the “energy powerhouse” of every cell which highly needs oxygen, mitochondria were very vulnerable to ischemia. Following cerebral ischemia, astrocytes transfer mitochondria to neurons for emergent energy supplement [22]. This observation inspired mitochondria transplantation as a promising therapy for various ischemic diseases, such as ischemia-infusion-induced heart and kidney injury [23, 24]. So far, as we know, only two groups examined the effects of exogenous mitochondria on ischemic stroke by using muscle or mesenchymal stem cell-derived mitochondria and reported reduced infarction size and decreased astrocytic and microglial activation [25, 26]. The very short *in vitro* and extracellular life span of mitochondria may limit the application of mitochondrial transplantation on oligodendrocyte degeneration and remyelination, which persists long after ischemia. However, our data showed exogenous mitochondria could be efficiently endocytosed by OPCs. Internalized mitochondria may either fuse with endogenous mitochondria or function independently. As the life span of mitochondria is about 2-4 weeks in cytoplasm, therefore, it is feasible to use mitochondrial transplantation to rescue energy failure in ischemia-attacked OPCs.

During development, OPCs require active mitochondria for proliferation and subsequent myelin formation ([11, 27]. Our data that NG2-positive and CNPase-positive cells internalize exogenous mitochondria reflected the metabolic requirement of OPCs. The increase of proliferation was consistent with the high energy requirement by OPCs and the boosting of energy supply by exogenous mitochondria. In detail, exogenous mitochondria may restore oxidative phosphorylation, thereby increasing ATP production. In addition, exogenous mitochondria may enhance the buffering of intracellular Ca2+ signaling, which helps to stop the activation of apoptotic signaling cascade promoted cell survival [28].

Interestingly, our data showed that exogenous mitochondria stimulated the expression of multiple lipid synthetic genes. As myelin is extremely lipid rich, and recent studies reported requirement of fatty acid and cholesterol synthesis for oligodendrocyte differentiation and myelination [19, 29], it is possible that exogenous mitochondria may steer lipid metabolism towards the direction more favorable for myelin formation [30]. How exogenous mitochondria rebalanced the lipid metabolism is worthy to be further investigated. In the present study, we cannot exclude other cells which internalized mitochondria may also exert beneficial effects. Nevertheless, our data provided a novel strategy for promoting myelination in ischemic stroke and other neurological disorders.

## Data Availability

The data used to support the findings of this study are included within the article.
